# Spatial and temporal conversion of nitrogen using *Arthrobacter* sp. 24S4–2, a strain obtained from Antarctica

**DOI:** 10.3389/fmicb.2023.1040201

**Published:** 2023-02-15

**Authors:** Yixuan Liu, Yumin Zhang, Yudi Huang, Jingjing Niu, Jun Huang, Xiaoya Peng, Fang Peng

**Affiliations:** ^1^China Center for Type Culture Collection (CCTCC), College of Life Sciences, Wuhan University, Wuhan, China; ^2^State Key Laboratory of Virology, Wuhan Institute of Virology, Center for Biosafety Mega-Science, Chinese Academy of Sciences, Wuhan, China

**Keywords:** *Arthrobacter*, aerobic DNRA, nitrogen storage, vesicle structure, spatial and temporal transformation of nitrogen, Antarctica

## Abstract

According to average nucleotide identity (ANI) analysis of the complete genomes, strain 24S4–2 isolated from Antarctica is considered as a potential novel *Arthrobacter* species. *Arthrobacter* sp. 24S4–2 could grow and produce ammonium in nitrate or nitrite or even nitrogen free medium. Strain 24S4–2 was discovered to accumulate nitrate/nitrite and subsequently convert nitrate to nitrite intracellularly when incubated in a nitrate/nitrite medium. In nitrogen-free medium, strain 24S4–2 not only reduced the accumulated nitrite for growth, but also secreted ammonia to the extracellular under aerobic condition, which was thought to be linked to nitrite reductase genes *nirB, nirD,* and *nasA* by the transcriptome and RT-qPCR analysis. A membrane-like vesicle structure was detected in the cell of strain 24S4–2 by transmission electron microscopy, which was thought to be the site of intracellular nitrogen supply accumulation and conversion. This spatial and temporal conversion process of nitrogen source helps the strain maintain development in the absence of nitrogen supply or a harsh environment, which is part of its adaption strategy to the Antarctic environment. This process may also play an important ecological role, that other bacteria in the environment would benefit from its extracellular nitrogen source secretion and nitrite consumption characteristics.

## 1. Introduction

The biosynthesis of key cellular components within cells requires nitrogen, which is one of the elements required for the survival of all living organisms. Only nitrogen-fixing bacteria and archaea can use nitrogen for growth; other organisms must rely on other nitrogen sources, such as nitrate and ammonium salts (Kuypers et al., 2018). The utilization of nitrate includes both assimilation and dissimilation, where nitrate is reduced to nitrite, and nitrite is eventually reduced to ammonium or nitrogen (Huang et al., 2020). The ammonium produced in assimilation is further synthesized into glutamine, and the ammonium produced in heterotrophy is released into the environment (Zhao et al., 2018). So far, the assimilation process in heterotrophic organisms has been insufficiently studied, and existing studies have focused on sequencing the genes encoding nitrate assimilation reductases in different microorganisms (Berube et al., 2015).

The fate of nitrite is an important boundary that determines the pathway of nitrate dissimilatory reduction in the environment, thus the nitrate dissimilatory process is divided into denitrification and dissimilatory nitrate reduction to ammonium (DNRA). Denitrification is a microbially mediated anaerobic transformation of nitrate to inert N2 gas, is the major removal pathway of nitrogen. In contrast, DNRA is a microbially mediated anaerobic pathway that retains bioavailable nitrogen within the ecosystem, using nitrate and producing ammonium (Friedl et al., 2018; Pandey et al., 2020). DNRA can be divided into fermentative and respiratory types with the reductases *Nap*/*Nrf* or *Nar*/*Nir* (Simon and Klotz, 2013; Wang et al., 2019). It has been shown that *NirB* and *NirD* have both nitrate assimilation dissimilation, so the nrfA gene is considered as a marker gene for the DNRA process (Fischer et al., 2012; Akhtar et al., 2013; Oliveira et al., 2013; Li X. F. et al., 2020). And the roles played by *nir*BD gene in nitrate assimilation and DNRA process need further study. Facultative and obligatory fermentative bacteria (*Clostridium*, *Bacillus*, and most *Enterobacter*) are the primary microorganisms responsible for DNRA processes in soils (Caskey and Tiedje, 1979). DNRA are currently found in different terrestrial ecosystems, such as agricultural soils, marine sediments and wastewater (Bernard et al., 2015; Heo et al., 2020; Li Z. et al., 2020).

The majority of the Antarctic region has been covered by snow and ice, limiting the activities of most organisms due to extreme conditions such as high solar radiation, low ice-free soil, and low nutrient levels (Ugolini and Bockheim, 2008; Adlam et al., 2010). Typically, Antarctic nitrogen sources exist in the form of nitrate and ammonium salts and at low concentrations of 0.2–15 μM and 0.03–20 μM (Wolff, 2013; Monteiro et al., 2020). Therefore, the nitrogen cycle is important for microbial activity in the Antarctic. Since these microorganisms account for a large fraction of the total biomass, they play an important role in the elemental cycling of the Antarctic ecosystem (Pointing et al., 2009; Chown et al., 2015). Most of the available nitrogen sources in Antarctica are produced by cyanobacteria and microorganisms through nitrogen fixation or degradation of organic nitrogen (Chen et al., 2021; Liu et al., 2021). Among them, the strategies and interactions of microbial adaptation to nitrogen depletion have become a hot topic of research (Cowan et al., 2014; Malard et al., 2019; Monteiro et al., 2020). The study on functional activity of soil nitrogen cycle microbes in Felders Peninsula of Antarctica found that denitrify activity of nutrient-poor soils in hilly slope were higher than soils occupied by animals and plants (Liu et al., 2017). The denitrify activity of the soil was detected by testing the nitrite production. However, both DNRA and denitrification can reduce nitrate to nitrite, this method is impossible to distinguish the two types of nitrogen metabolism: removes fixed nitrogen or retains it within the ecosystem. it is possible that the detected nitrite produced by both denitrification and DNRA. However, it is generally accepted that DNRA action exists in low oxygen environments, and the marker gene *nrf*A was also not detected in these soils. Perhaps it is due to imperfect indicator genes and biochemical assays that the process of nitrate reduction to ammonia has yet to be detected in Antarctica.

*Arthrobacter* is the dominant genus in Antarctica, particularly in some glacial regions (Strauss et al., 2012). In *Arthrobacter*, genomic results revealed the widespread presence of genes related to sigma factors, signal transduction pathways, nitrification, denitrification, and genes induced by cold shock, oxidation, and osmotic stress (Strauss et al., 2012; Dsouza et al., 2015). Most studies have shown that *Arthrobacter* is involved in nitrification and denitrification, which is undoubtedly harmful to the nutrient-depleted Antarctic (Strauss et al., 2012; Dsouza et al., 2015; Zhang et al., 2020; Liu et al., 2021). It was also discovered that *Arthrobacter* performed DNRA in anaerobic culture (Eschbach et al., 2003). However, it is unknown whether Antarctic *Arthrobacter* can produce ammonia, and if so, *Arthrobacter* will play a larger role in the Antarctic nitrogen cycle. Moreover, the DNRA-associated microorganisms and their ecological roles are unknown in the Antarctic.

Strain 24S4–2 was isolated from a moraine on Antarctica’s Collins Ice Cap and shown to be capable of generating ammonia and nitrite. We suspected that this strain may have been capable of nitrate reduction under aerobic conditions and not denitrification as commonly considered. Thus, we investigated the nitrogen metabolism characteristics of strain 24S4–2 for ammonia production and nitrogen-free growth to explore its adaptation mechanism and possible ecological role in the extreme Antarctic environment. Therefore, we proposed the following hypotheses for the nitrate utilization process of strain 24S4–2: i. nitrate utilization by strain 24S4–2 could belong to the heterotrophic nitrate pathway, ii. under the condition of sufficient external nitrogen source, strain 24S4–2 could accumulate nitrogen source intracellularly and be used for its growth and development during nitrogen starvation, iii, the accumulated nitrogen source may be stored in a separate space, avoiding toxic effects for the cells, while providing an anaerobic environment for the DNRA process. The study provides new insights into the role and ecological status of *Arthrobacter* in extreme environments.

## 2. Materials and methods

### 2.1. Site description and sampling

In Fields Peninsula, Antarctica, a moraine sample was collected from the edge of the Collins Ice Cap (62°10.938′S 58°51.999’W). The strain 24S4–2 was isolated using the conventional dilution-plating approach R2A medium plates and cultured at 4°C for 2 weeks. Individual colonies on these plates were purified by transferring them to other plates and incubating them at 20°C for 4 days. This strain was routinely cultivated at 20°C on R2A slants and lyophilized for storage.

### 2.2. Culture medium and culture conditions

In a shaker, strain 24S4–2 was incubated in R2A liquid medium for 2 days at 20°C and 160 rpm. It was then inoculated into the dissimilatory nitrate reduction to ammonium (DNRA) basal medium, which contained 4 g KH_2_PO_4_, 7 g K_2_HPO_4_, 5.13 g CH_3_COONa, 0.5 g MgSO_4._7H_2_O, 0.05 g FeCl_2._7H_2_O, and different nitrogen sources (30 mM NaNO_3_ or 30 mM (NH_4_)_2_SO_4_ or 30 μM NaNO_3_), and incubated for 7 days. Alternately, it was inoculated into a denitrification basal medium, which contained 5.44 g KH_2_PO_4_, 10.49 g K_2_HPO_4_, 3.28 g CH_3_COONa, 0.05 g MgSO_4_.7H_2_O, 0.005 g MnSO_4._4H_2_O, 0.125 mg FeSO_4_.7H_2_O, 0.5 mg CaCl_2,_ and different nitrogen sources (30 mM NaNO_3_ or 30 mM (NH_4_)_2_SO_4_ or 30 μM NaNO_3_).

### 2.3. The nitrogen transformation capacity of strain 24S4–2

To investigate the nitrogen metabolic pathway of strain 24S4–2, the strain was cultured in different nitrogen source media and its metabolites were examined. A single colony was inoculated into a 5 mL R2A liquid medium to enrich the 24S4–2 strain for denitrification and intracellular nitrate analysis at 160 rpm/20°C for 48 h. The pellet was then washed three times with sterilized water after centrifuging the pre-cultured 24S4–2 strain for 15 min at 10000 rpm. The final pellet was incubated at 20°C under 160 rpm in a basal medium containing 30 mM NaNO_3_ or NaNO_2_. Using HPLC (Ultimate 3,000, Dionex), nitrate and nitrite concentrations were measured every 12 h to probe the DNRA of strain 24S4–2. In order to study the presence of nitrate in the intracellular liquid, strain 24S4–2 was cultivated for 3 days before being centrifuged at 10000 rpm for 15 min and rinsed three times with sterilized water. The pellet was suspended in 30 mL sterile water and was disrupted 5 times by a high-pressure homogenous cell disruption instrument (FPG 12800, SFP) until the liquid became clear.

An Agilent Eclipse XDB-C18 column (4.6 × 250 mm, 5 μm) was used to separate the samples. The mobile phase consisted of 100% HPLC-grade methanol and 1.25 mM mixed phosphate (containing 3 mM tetrabutylammonium bromide) in a 15:85 ratio, with a column temperature set at 30°C, 230 nm detection wavelength, 20 μL injection volume, and 1.0 mL/min flow rate. A standard curve was used to calculate the nitrate concentrations.

The Griess reaction was used to examine nitrite. The ammonium concentration was evaluated using Nessler’s reagent method (absorption at a wavelength of 420 nm).

### 2.4. Microstructure observation of strain 24S4–2

In order to investigate the potential positions of intracellular nitrogen sources in strain 24S4–2, ultrathin sections were made of strain 24S4–2 and its internal structure was observed by transmission electron microscopy. Strain 24S4–2 was embedded in propylene oxide/supr resin and polymerized for 48 h at 60°C in an oven. The samples were then delivered to the transmission electron microscopy sharing platform to be prepared ultrathin sectioning machine. On a 300-mesh copper grid (BZ11022b; Zhongjing Science and Technology, Beijing, CN), 10 μL of the suspension were covered with produced carbon films and incubated for 1 min. The filter paper was used to remove the excess suspension from the edges of the grid. The grids were negatively stained with 10 μL of 1% uranyl acetate for 1 min, and the excess staining solution was removed with filter paper. The samples were examined using a Transmission Electron Microscope (JEOL, JEM-1400plus, Japan) after drying for 10 min. The internal parts of strain 24S4–2 were analyzed using transmission electron microscopy JEM-2010 FEF (Nippon Optical Limited).

### 2.5. Growth activity at different oxygen concentrations

Nitrate reduction by dissimilatory processes usually occurs under anaerobic conditions. To further investigate the effect of oxygen on nitrate reduction by strain 24S4–2, strain 24S4–2 was incubated in the same medium under different oxygen concentrations and its growth and ammonium production were observed. To ensure that oxygen is removed, a sterile basal medium containing 30 mM nitrate and Cyrillic flasks (7 mL) were placed in an anaerobic workstation (AW400TG, ELECTOROTEL) 2 days in advance. 24S4–2 was incubated in R2A medium for 2 days before being centrifuged at 12,000 rpm for 15 min at 4°C, and the supernatant was discarded. The pellet was then washed three times with sterile water before being discarded. The organisms were mixed thoroughly to the medium in an anaerobic workstation, and 2 mL of the bacterial solution was added to each cilium vial and capped with a sealing stopper. All the air from the vial was removed using a 5 mL syringe, and then 5 mL argon was added, and the corresponding volume of argon was removed according to different oxygen concentrations, and finally, equal volumes of oxygen, set 0, 5, and 20% oxygen concentrations, respectively were added. The ammonium content in the medium was tested using five replicates of each experiment incubated at 20°C for 3 days.

### 2.6. Whole-genome sequencing

A genomic DNA extraction kit (QIAGEN Cat. No. Q13343) was used to isolate the genomic DNA of strain 24S4–2. The genomic DNA was detected using 0.75% agarose gel electrophoresis and accurately quantified using Qubit. The whole-genome sequencing was done at Nextomics Bioscience Co., Ltd., Wuhan, China, on the PacBio RS II platform. The Covaris g-TUBE was used to randomly interrupt the genomic DNA of strain 24S4–2; magnetic beads were used to concentrate and purify the large DNA fragment, and the stem-loop sequencing link was attached to both ends of the DNA fragment sequencing adapters. Nextomics Biosciences Co., Ltd. produced a 20-kb library from genomic DNA isolated from strain 24S4–2. After that, Hierarchical Genome Assembly Process 3 (HGAP3) was used to perform the initial assembly (Chin et al., 2013). On the PacBio RS II platform, a total of 85,457 reads with an average length of 5,207 bp from one SMRT cell were sequenced. Celera assembler put together all of the high-quality paired readings (Miller et al., 2008). GenBank/EMBL/DDBJ contains the full genomic sequences of strain 24S4–2. The DNA G + C content of strain 24S4–2 was determined according to the genomic DNA sequence. The tRNA scan-SE 1.23 software (Lowe and Eddy, 1997) was used to predict tRNA, and the RNAmmer 1.2 prediction software was used to predict rRNA (Lagesen et al., 2007). The average nucleotide identity (ANI) value between strain 24S4–2and the reference strain was calculated using the OrthoANI analysis with OAT software (Lee et al., 2016). The Gene Ontology (GO), Kyoto Encyclopedia of Genes and Genomes (KEGG) (Du et al., 2014), Non-Redundant Protein (NR), Clusters of Orthologous Group (COG) (Tatusov et al., 2001; Jensen et al., 2008), and Swiss-Prot (Magrane and UniProt, 2011) databases were also used to predict gene functions using BLASTp and the same BLAST thresholds.

### 2.7. Analysis of the transcriptome associated with nitrate or nitrite reduction

To further explore the genes involved in the nitrate or nitrite reduction of strain 24S4–2 in nitrogen-free and nitrogen-free conditions, the transcriptome of strain 24S4–2 was sequenced. Strain 24S4–2 was cultured for 114 h before being centrifuged at 12000 rpm for 20 min at 4°C, and the supernatant was discarded. The precipitate was suspended in 50 mL of nitrogen-free medium and ddH_2_O in equal parts, centrifuged at 12000 rpm for 20 min at 4°C, and the supernatant was discarded. Bacteria were placed in an RNase-free mortar and ground with liquid nitrogen until the bacteria were fully pulverized. With three replicates per sample, the Bacterial RNA kit (R6950, OMEGA) was used to extract bacterial total RNA. The transcriptome was evaluated using the Megabio Cloud platform after the entire RNA was delivered to Megabio for RNA sequencing. The Illumina sequencing platform was used to sequence the transcriptome of *Arthrobacter* sp. 24S4–2. The transcriptomic data were subjected to parametric analysis using 24S4–2 as a reference genome.

RT-qPCR of RNA from strain 24S4–2: RNA was extracted after incubating bacteria in different conditions. The whole cDNA was then generated using a PrimeScriptTM RT Master Mix according to the manufacturer’s instructions (Perfect real-time Takara). The three replicates of a single real-time PCR experiment were used to determine the transcription level of three identified genes involved in nitrite reduction. A TB Green® premix Ex TaqTM (Tli RNaseH Plus Takara) was used to make each RT-qPCR mixture. A CFX96 Real-Time PCR Detection System (Bio-Rad) was used for the Rt-qPCR. The qPCR cycling protocol was set as follows; 95°C for 30 s, followed by 39 cycles of 95°C for 5 s, and 60°C for 30 s. The transcription level of 50S ribosomal L13 was employed as an internal control because this is a single-copy gene. All the primers for the three genes are listed in Supplementary Table S1.

## 3. Result

### 3.1. Isolation and characteristic of strain 24S4–2

Strain 24S4–2, a heterotrophic-aerobic nitrate dissimilation and reduction to ammonium bacteria, was isolated from the Collins Ice Cap moraine on Fields Peninsula, Antarctica (62°10.938′S 58°51.999’W). The 24S4–2 strain is a Gram-positive, short rod-shaped bacterium with an approximate size of 0.2 μm × 0.4 μm. The 16S rRNA gene sequence (GenBank ON005313) of strain 24S-2 with a nearly full length of 1,400 bp was obtained and found to have the highest similarity with *Arthrobacter oryzae* at 99.1% (Supplementary Figure S1). In order to validate distinct species status for strain 24S4–2, the ANI value of genome-wide comparisons between the strain 24S4–2 and its closely related phylogenetic neighbours sharing more than 98% 16S rRNA similarity. Genome-wide analysis showed that strain 24S4–2 had the highest ANI value of 80.1% with *Arthrobacter globiformis* among similar strains. Although the 16S similarity between strain 24S4–2 and *A. oryzae* was 99.1%, and the ANI value between them was 78.45% (Supplementary Figure S1). The ANI threshold for species discrimination were 95–96% (Meier-Kolthoff et al., 2013; Kim et al., 2014). It could be a novel species of the genus *Arthrobacter* (*Arthrobacter* sp. 24S4–2).

The whole-genome sequence of strain 24S4–2 has been deposited in the NCBI database (GenBank CP040018). The whole genome of *Arthrobacter* sp. 24S4–2 comprises a circular chromosome with a GC content of 65.07% and a length of 5,563,753 bp. In total, 5,592 genes were predicted on the chromosomes, with 5,564 functionally assigned protein-encoding genes and the remaining genes as hypothetical proteins. The rapid annotation using subsystems technology (RAST) server was used to annotate the whole genome of strain 24S4–2 genetically. Allantoin utilization (9 genes), nitrosative stress (1 gene), nitrate and nitrite ammonification (10 genes), ammonia assimilation (10 genes), and denitrification (5 genes) are all involved in nitrogen metabolism. Supplementary Figure S2 shows a map of the nitrogen metabolism pathway, with red boxes indicating genes in the genome of strain 24S4–2 that are believed to be involved in nitrogen metabolism. The genes involved in the dissimilatory nitrate reduction to ammonium (DNRA) pathway include *narG*, *narH*, *narI*, *nirB,* and *nirD*. Nitrate reduction is linked to the genes *narG*, *narH,* and *narI*, while nitrite reduction is linked to the genes *nirB* and *nirD* (Elliott et al., 2004; Tang et al., 2019).

### 3.2. Ammonium production performance and nitrite reductase activity of strain 24S4–2

**Figure 1:**
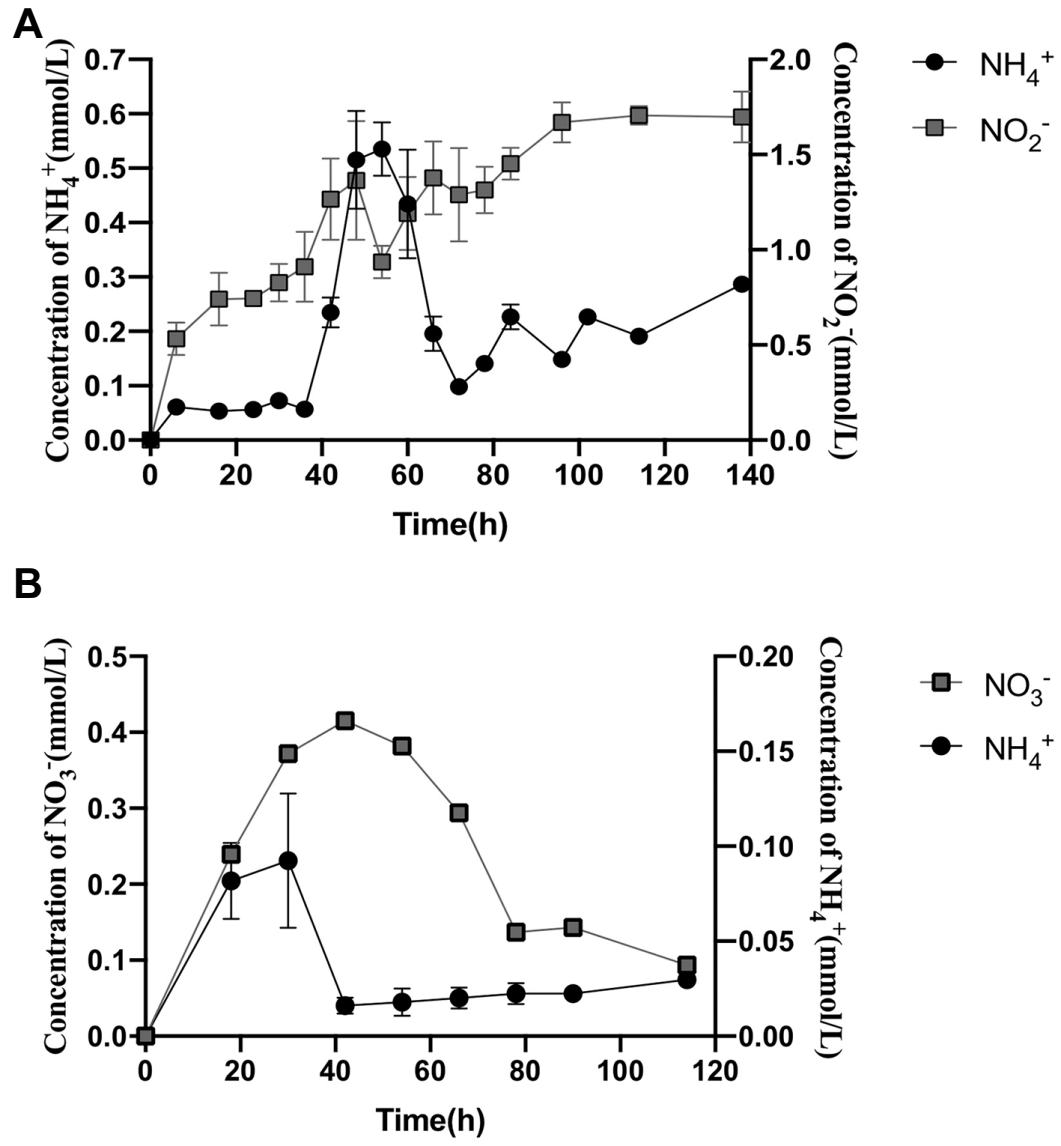
Extracellularly produced nitrogen compounds and contents by strain 24S4–2 grown solely in nitrate or nitrite. **(A)** extracellular nitrite and ammonium concentrations with 30 mM nitrate as the sole source of nitrogen; **(B)** extracellular nitrate and ammonium concentrations with 3 mM nitrite as the sole source of nitrogen.

Strain 24S4–2 was inoculated into MM medium with different nitrogen sources to understand the types of nitrogen sources available better. The sole nitrogen source used were 30 mM ammonium sulfate, 30 mM sodium nitrate, and 3 mM sodium nitrite. Strain 24S4–2 could grow with each of the three nitrogen sources separately (Supplementary Figure S3), with ammonium sulfate growing the fastest, followed by nitrate and nitrite. For strain 24S4–2, the nitrite tolerance concentration was 20 mmol/L (Supplementary Figure S4). Strain 24S4–2 barely developed in 20 mM nitrite media. When the nitrite content was 3 mmol/L, strain 24S4–2 developed the fastest. In the medium containing 30 mM nitrate as the sole nitrogen source, strain 24S4–2 reduced nitrate to nitrite and ammonium (Figure 1). Notably, the extracellular ammonia concentration peaked at 0.533 mmol/L at 54 h, while the extracellular nitrite concentration continued to increase and peaked at 1.69 mmol/L at 114 h.

Nitrite was gradually depleted in the medium with nitrite as the sole nitrogen source, but nitrate and ammonium were detected and reached 0.42 mmol/L at 42 h and 0.611 mmol/L at 30 h, respectively (Figure 1). In the nitrate reduction process, nitrite reductase (*NiR*) was a critical enzyme. The specific activity of *NiR* was expressed as nanomoles of ammonium/min/mg of proteins (nmol/min/mg). When nitrate was the only nitrogen source, the specific activity of nitrite reductase *NiR* was 0.0642 U/mg protein under aerobic conditions. The enzymatic activity of strain 24S4–2 nitrite reductase was also detected in the medium with ammonium as the sole nitrogen source; however, it was only 0.0036 U/mg protein; thus, its activity can be ignored. The findings suggested that the intracellular nitrite reductase of strain 24S4–2 was involved in the 114 h incubation in a nitrate environment.

Based on these findings, we hypothesize that strain 24S4–2 can reduce nitrate to nitrite and thereafter to ammonium, i.e., DNRA activity. DNRA, on the other hand, is carried in under anaerobic conditions, whereas this strain does not grow in anaerobic conditions and produces ammonium in aerobic ones. As a result, we investigated the impact of various oxygen levels on this action. Supplementary Figure S5 shows no significant variation in nitrite production at different oxygen concentrations. All could reach 0.8 mmol/L, while ammonium was identified at a higher level of 0.11 mmol/L when incubated at 20% oxygen concentration. Supplementary Table S2 shows that at an oxygen concentration of 20%, the OD_600_ of the strain changed from 0.1 to 0.2 (Supplementary Table S3). At oxygen concentrations of 0 and 5%, strain 24S4–2 scarcely developed (Supplementary Table S3). Because the experiment was incubated in resting condition, it resulted in a delayed growth of strain 24S4–2. The ammonium concentration was maximum at a 20% oxygen concentration because of the increased bacterial abundance. It is worth noting that strain 24S4–2 may reduce nitrate to nitrite and ammonium in anaerobic conditions, while strain 24S4–2 cannot be grown in anaerobic conditions. Conversion of nitrate to ammonium by strain 24S4–2 differed depending on the partial pressure of oxygen.

### 3.3. Ammonium production performance of strain 24S4–2 without nitrogen sources

To investigate whether strain 24S4–2 can develop in a nitrogen-free medium, it was initially inoculated into a medium containing ammonium, nitrate, or nitrite as the sole nitrogen source for 114 h before being put into a nitrogen-free medium. Strain 24S4–2 could grow in the nitrogen-free medium after incubation in nitrate or nitrite medium but not in the nitrogen-free medium after incubation in ammonium medium, as shown in Supplementary Figure S6.

**Figure 2:**
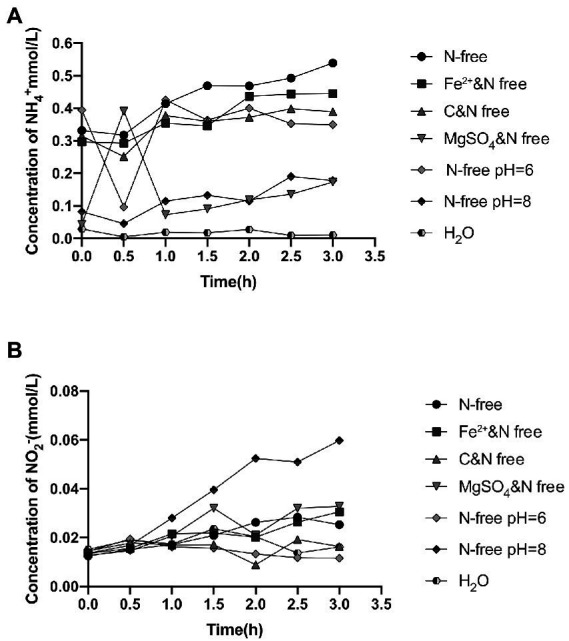
Extracellular ammonium and nitrite concentrations in different nitrogen-free media after 114 h of incubation with strain 24S4–2. **(A)** extracellular ammonium concentration and **(B)** extracellular nitrite concentration.

Strain 24S4–2, which grew for 114 h in nitrate media, was shown to be able to grow and detect ammonium extracellularly in a nitrogen-free medium but not in water. In nitrogen-free media, we anticipated that strain 24S4–2 could also perform DNRA. The components in the nitrogen-free media were removed one by one to understand the conditions of ammonia formation. Strain 24S4–2 generated ammonium without effect when the medium lacked nitrogen, ferrous ions, a carbon source, and dipotassium hydrogen phosphate. The extracellular ammonia concentration dropped dramatically when the nitrogen-free medium was free of magnesium ions or alkaline (pH 8.0) (Figure 2). However, only in an alkaline medium (pH 8.0) did the extracellular nitrite concentration increase significantly, presumably due to the inhibition of nitrite reductase in an alkaline environment, resulting in nitrite accumulation. A paucity of magnesium ions may have hampered the release of nitrite from the cell.

### 3.4. Intracellular accumulation and transformation of nitrate and nitrite

**Figure 3:**
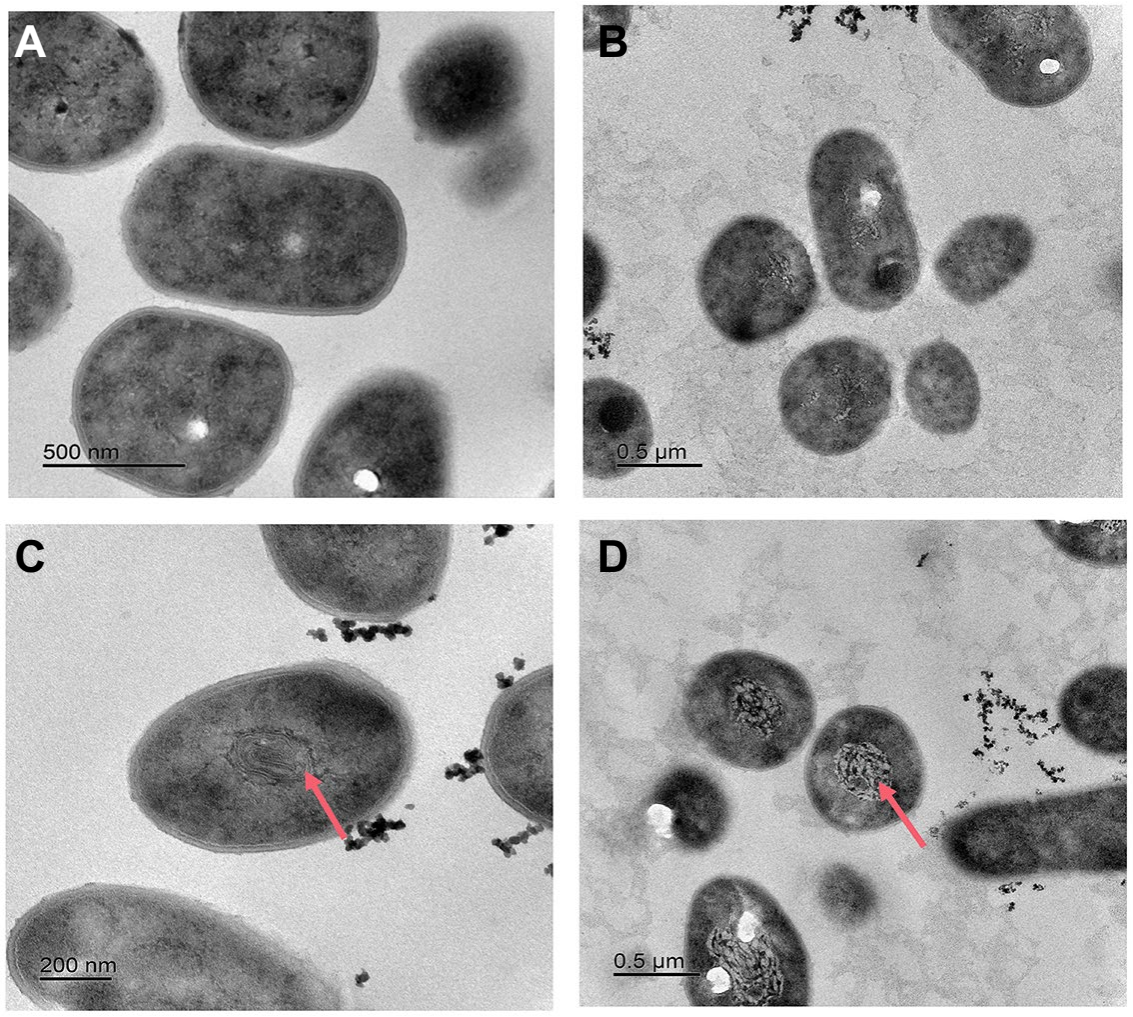
Transmission electron micrographs of strain 24S4–2 in various mediums. **(A)** R2A medium, **(B)** ammonium as the sole nitrogen source medium, **(C)** nitrate as sole nitrogen source medium, and **(D)** nitrite as sole nitrogen source medium.

After incubation in nitrate or nitrite medium, strain 24S4–2 may continue to thrive nitrogen-free but not in ammonium. In this experiment, four media were employed to grow strain 24S4–2, namely R2A, ammonium medium, nitrate medium, and nitrite medium, to observe how varied nitrogen source cultures affected its internal structure. Strain 24S4–2 was cut into ultra-thin sections after 5 days of incubation, and its internal structure was examined using transmission electron microscopy (Figure 3). When nitrate or nitrite was employed as the sole carbon source, a membrane-like vesicle structure (200–500 nm) was generated inside strain 24S4–2, but this structure was not detected when strain 24S4–2 was inoculated into R2A and ammonium as the sole nitrogen supply media. Furthermore, this vesicle swelled larger when nitrite was employed as a nitrogen supply instead of nitrate. Transmission electron microscopy JEM-2010-FEF was used to analyze the elements in ultrathin sections of 24S4–2. The concentration of nitrogen was found to be relatively high in the region of vesicle-like structures in the nitrate medium. Only N and O elements were examined, and N accounted for 65.6% of the atomic ratio, owing to the accumulation of nitrogen sources (Supplementary Figure S7A). The percentage of N elements in other locations was only 23.6%. However, because the three elements, C, N, and O were close to each other and difficult to differentiate, it was difficult to distinguish the peaks of nitrogen elements (Supplementary Figure S7).

**Figure 4:**
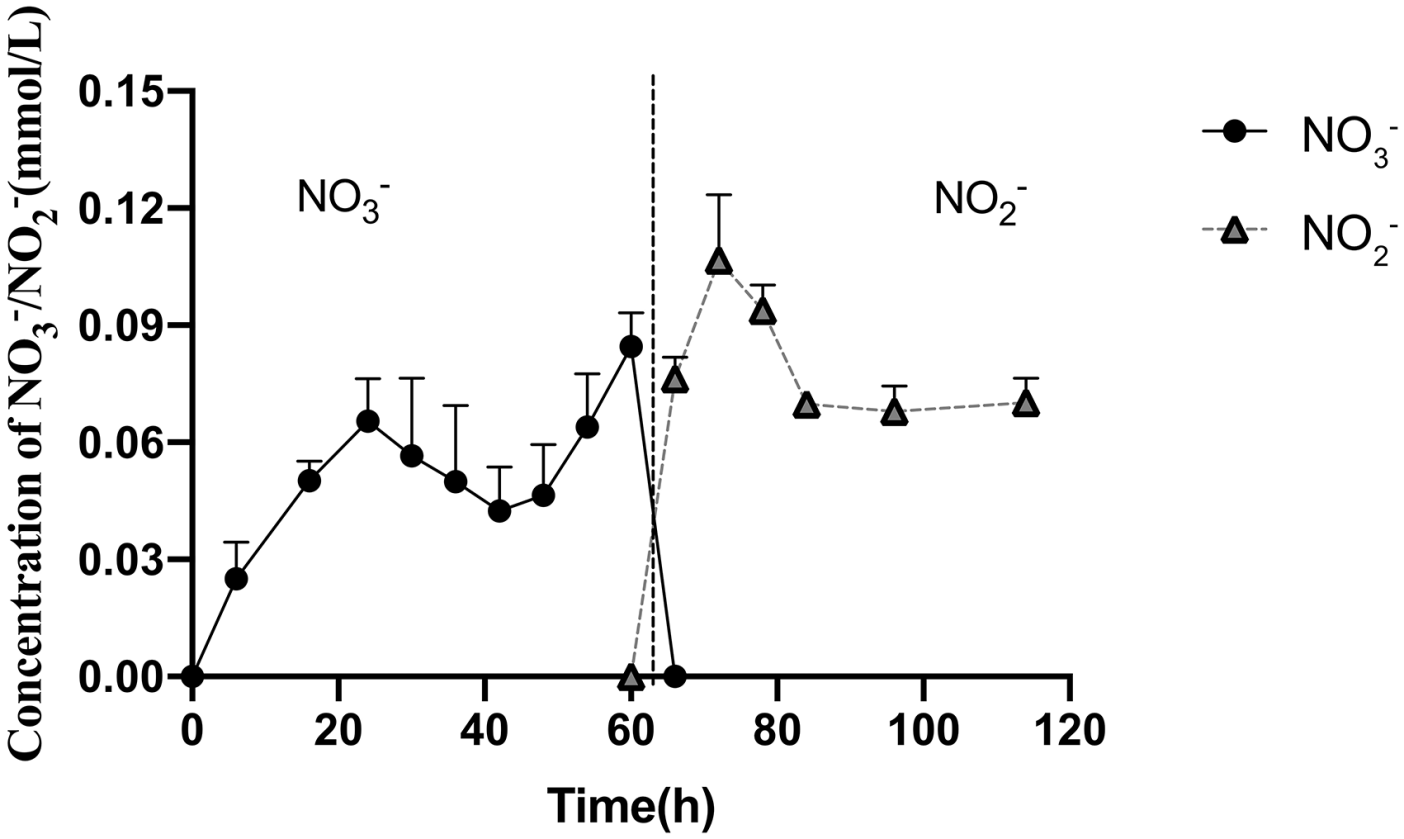
Strain 24S4–2 accumulated nitrogen intracellularly. Nitrate was accumulated intracellularly before 60 h, and nitrite was accumulated thereafter.

Based on previous results that strain 24S4–2 could grow in a nitrogen-free medium and create vesicles in the cell, we speculated that when nitrate and nitrite were present in the external environment, strain 24S4–2 would accumulate nitrogen sources in the cell. To test our hypothesis, strain 24S4–2 was inoculated with nitrate as the sole nitrogen source and then fragmented the cells and determined the internal nitrogen source type and content. The results revealed that nitrate was accumulated intracellularly during the early stages of growth (Figure 4), and the concentration grew progressively over time, peaking at 0.09 mmol/L after 54 h. The intracellularly accumulated nitrate was converted to nitrite after 60 h, and the intracellular nitrite concentration remained constant at 0.09 mmol/L. The vesicle was 200 nm x 500 nm in size. When viewed as a cylinder, the internal concentration of nitrogen accumulated by strain 24S4–2 is 10,000 times higher than that in the external environment, as shown in Figure 3. The findings supported our hypothesis that strain 24S4–2 could accumulate nitrate and nitrite and that the nitrate accumulated might be converted to nitrite. Furthermore, we hypothesized that the nitrogen source accumulated in the vesicle structure might be stored.

**Figure 5:**
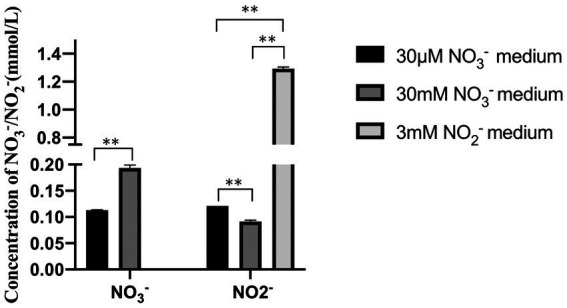
Intracellular accumulation of nitrogen sources by strain 24S4–2 in different nitrogen source media. Types and concentrations of intracellularly accumulated nitrogen sources of strain 24S4–2 cultured for 20 d in 30 μM nitrate medium, 30 mM nitrate medium and 3 mM nitrite medium, “*” represents value of *p* < 0.05, “**” represents value of *p* < 0.01, and NS: Not Significant.

Even when the nitrate concentration in the media was just 30 μM, intracellular nitrate, and nitrite accumulated, as shown in Figure 5. When nitrite was the main nitrogen source, nitrate did not accumulate intracellularly, and it is worth noting that significantly more nitrite accumulated intracellularly under nitrite conditions than nitrate and nitrite under nitrate conditions. This process could explain why the vesicles swelled larger in a condition where nitrite is the only supply of nitrogen. Only nitrite was identified intracellularly at a concentration of 1.29 mmol/L under the 3 mM nitrite condition. The above experiments revealed that strain 24S4–2 has a higher intracellular potential for nitrite accumulation than nitrate.

### 3.5. Nitrate reduction process-relevant genes

**Figure 6:**
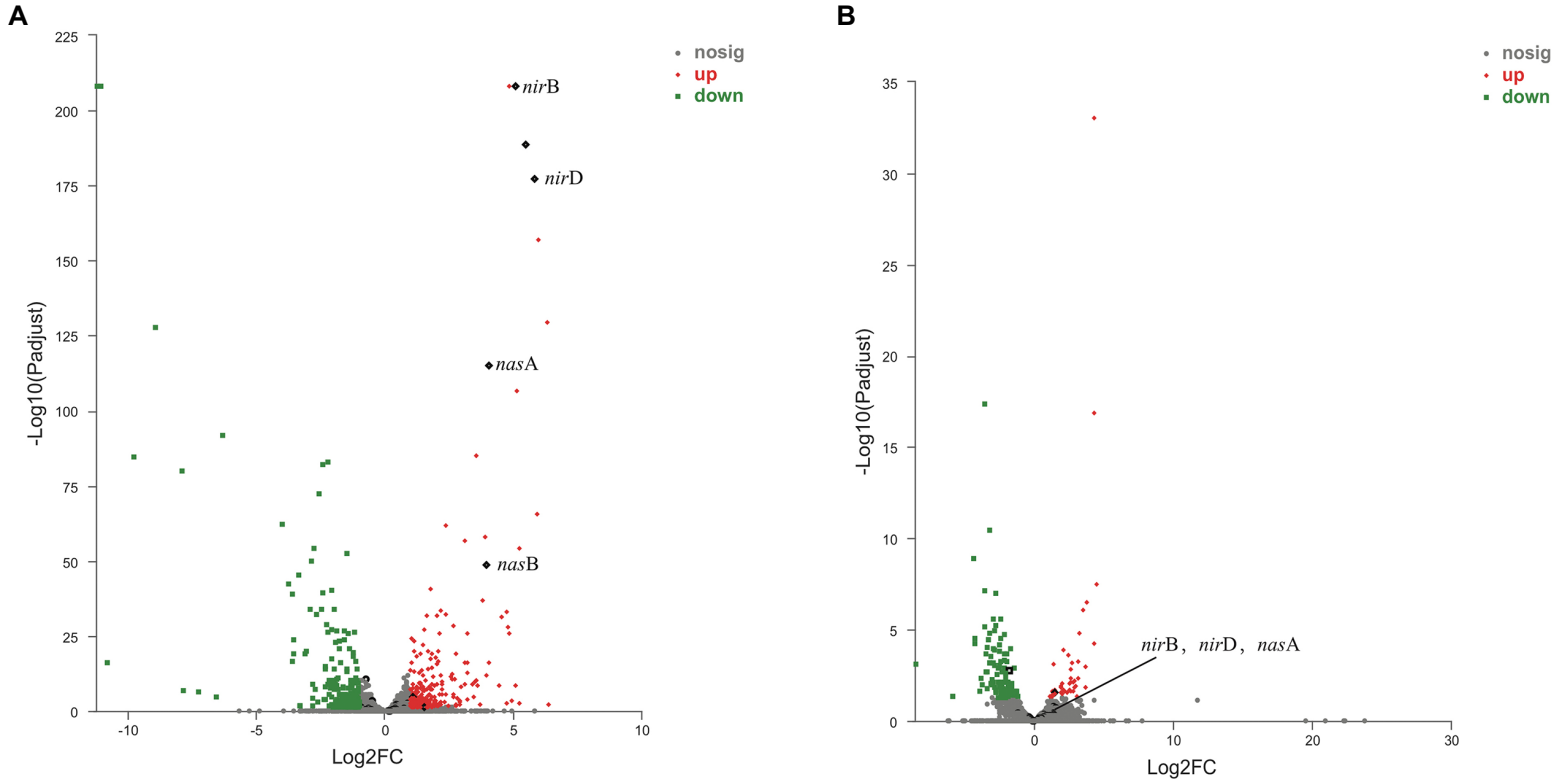
Volcano plot of the difference in gene expression between strain 24S4–2 in nitrate medium and ammonium medium (control). **(A)** Volcano plot of the difference in gene expression between strain 24S4–2 in nitrate medium and ammonium medium (control), **(B)** Volcano plot of differential gene expression of strain 24S4–2 in nitrogen-free medium and water (control) after 114 h incubation in nitrate medium. Red points indicate up-regulated expression genes; green points indicate down-regulated expression genes; black points indicate DNRA pathway-related genes.

RNA-Seq was used to compare the transcriptional profiles of strain 24S4–2 cultivated under different conditions. DNRA pathway-related genes are found in the up-regulated fields of a volcano plot of the gene with expression level modified, strain 24S4–2 cultured on nitrate medium and ammonium medium (control). The *narG, nirB, nirD,* and *nasA* were among the expression genes that were significantly up-regulated (Figure 6A). After 114 h of incubation in nitrate medium, transcriptome data of strain 24S4–2 in the nitrogen-free medium and water revealed that these nitrate reduction-related genes were likewise elevated in the nitrogen-free medium. According to the results, transcripts of these genes were raised in the assimilation or dissimilation of nitrate to produce ammonia (Figure 6B).

**Figure 7:**
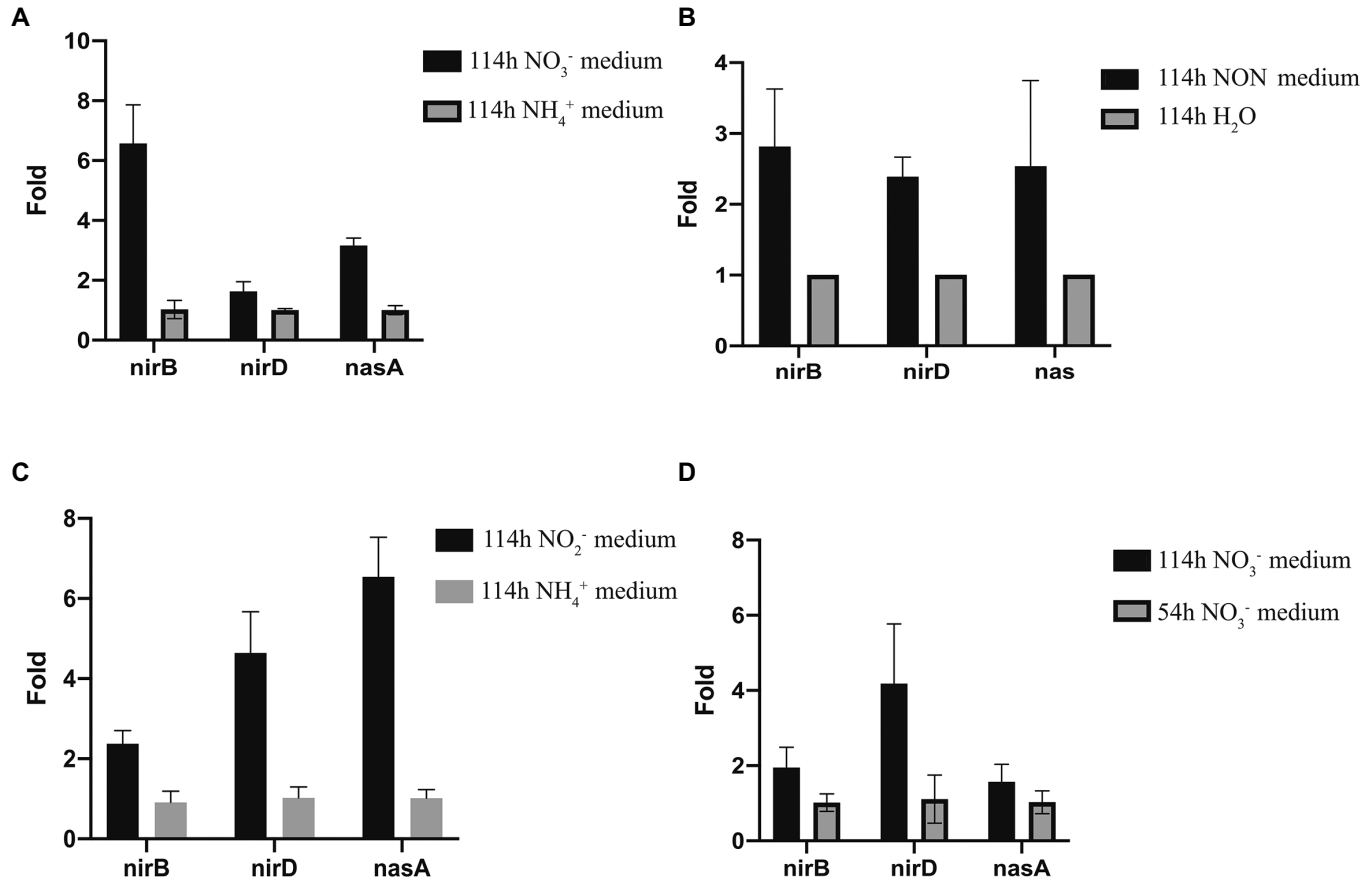
Quantitative PCR analysis of nirB, nirD, and nasA expression of strain 24S4–2 grow in a different mediums. **(A)** strain 24S4–2 grow in medium with nitrate or ammonium as the sole nitrogen source, **(B)** strain 24S4–2 grow in nitrogen-free medium (NON) and water, **(C)** strain 24S4–2 grow in medium with nitrite or ammonium as the sole nitrogen source, and **(D)** strain 24S4–2 grown in medium with nitrite as the sole nitrogen source for 114 h or 54 h.

RT-qPCR was used to analyze the nitrite reductase-related genes, *nirB, nirD,* and *nasA*, which were up-regulated in transcriptomic analysis. When strain 24S4–2 was cultured on nitrate media and ammonium (control) medium, RT-qPCR results showed that the expression levels of *nirB*, *nirD,* and *nasA* were increased, which was compatible with transcriptome data (Figure 7A). When strain 24S4–2 was inoculated into a nitrogen-free medium instead of ddH_2_O, the transcript levels of genes *nirB*, *nirD,* and *nasA* were up-regulated (Figure 7B). The transcriptome sequencing data corroborated these findings. Otherwise, as shown in Figure 7C, the expression of genes *nirB, nirD,* and *nasA* were up-regulated in the nitrite medium compared to in the ammonium medium, with gene *nasA* showing the highest upregulation. Remarkably, when strain 24S4–2 was cultured in nitrate media for 114 h versus 54 h, the genes *nirB*, *nirD,* and *nasA* were differentially up-regulated (Figure 7D). In 54 h culture, strain 24S4–2 produced more ammonium; however, the expression of gene *nasA* was lower. NasA may be involved in the conversion of nitrate stored intracellularly to nitrite.

## 4. Discussion

Although 16S rRNA analysis showed that strain 24S4–2 was 99% similar to *A. oryzae* DSM 25586^T^, the ANI value of both was 78.45%. The ANI value of strain 24S4–2 and another non-type strain of *A. oryzae* TNB02 (Cho et al., 2019) obtained from Antarctica was 79.26%. The combination of 16S similarity and ANI can be used in a systematic process to identify and confirm a novel species. ANI values are only calculated when the 16S similarity between species is higher than 98%. At this point, At this point, the ANI values between strain 24S4–2 and similar strains were below the species threshold <95–96% (Chun et al., 2018). Therefore, it indicated that the strain is a potential novel species of *Arthrobacter* (Meier-Kolthoff et al., 2013; Kim et al., 2014), but it still needs to be identified after several taxonomic tests such as physiological and biochemical tests.

Many members of the genus *Arthrobacter* have been found to have the ability to reduce nitrate, such as *Arthrobacter oryzae*, *Arthrobacter pokkalii*, *Arthrobacter globiformis*, *Arthrobacter arilaitensis* (Pinar and Ramos, 1997; Kageyama et al., 2008; He and Li, 2016; Krishnan et al., 2016). And it has been shown that the reduction of nitrate by *Arthrobacter* is usually considered to be through denitrification (He et al., 2017; Zhong et al., 2018; Cai et al., 2019). However, nitrate reduction to ammonium by *Arthrobacter* has rarely been reported. At present, the metabolisms known to produce ammonia are nitrogen fixation and DNRA, which are generally marked in the environment by the nitrogen fixation gene *nif* and the nitrite reductase gene *nrf* (specific to Gram-negative bacteria), respectively (Cannon et al., 2019; Wang et al., 2019; Mise et al., 2021). We did not find the similar two genes in the genome of strain 24S4–2, but this strain is able to produce ammonia from nitrate and nitrite and can even grow in nitrogen-free culture conditions. Acetylene reduction experiments determined that this strain cannot fix nitrogen but can accumulate nitrate and nitrite. Although intracellular nitrate was detected at low concentrations, strain 24S4–2, around 0.4 μm in size, accumulated intracellularly at a concentration 10,000 times higher than in the external environment. Small nitrite levels were observed to accumulate intracellularly in *A. arilaitensis* (He et al., 2020), but the accumulation and conversion of nitrate in *Arthrobacter* was first demonstrated. In 1995, it was shown that filamentous sulfur bacteria accumulate huge amounts of nitrite intracellularly and that *Thioploca* spp. filamentous monomers accumulated intracellular nitrate at concentrations up to 500 mM in their vesicles (Fossing et al., 1995; Hogslund et al., 2009). *Beggiatoa* spp., a large, vacuum- susceptible sulfur bacteria, can accumulate intracellular nitrate at concentrations of 130 to 160 mM (Mussmann et al., 2003; Hinck et al., 2007). *Beggiatoa* and *Thioploca,* two nitrate-accumulating bacteria, had similar morphologies, being filamentous and having huge vacuoles. When strain 24S4–2 accumulates nitrate and nitrite, electron microscopy studies revealed that it forms an internal vesicle formed of folded membranous components. However, when ammonium was supplied as the sole nitrogen, this structure did not form. To date, no known biochemical mechanism can explain the high concentration of nitrite observed in the intracellular fluid. Therefore, the metabolic mechanism of the strain 24S4–2 toward different nitrogen sources requires further study.

Strain 24S4–2 not only accumulated nitrate and nitrite intracellularly, but also subsequently converted nitrate to nitrite in the cell. Moreover, in nitrogen free medium, the accumulated nitrate and nitrite were reduced to ammonia and support the growth of Strain 24S4–2. It is considered that the strain has nitrate reduction ability and the nitrate/nitrite reductase activities and related genes (*NarG*/*NirB*) were detected. These may be the strategy of strain 24S4–2 to cope with nitrogen barren, that is, to assimilate the accumulated nitrate and nitrite in the cell to survive without nitrogen. Nitrate reduction can be divided into assimilatory nitrate reduction and dissimilatory nitrate reduction. Normally, assimilated nitrate reduction generally does not accumulate nitrite and release ammonia. It synthesizes amino acids through glutaminase, which has a high affinity for ammonia (Zhao et al., 2018; Huang X. J. et al., 2021). However, strain 24S4–2 not only assimilated the accumulated nitrite to grow, but also release ammonia outside the cell. The nitrite reductase *NirB* which detected in this process was reported to reduce nitrite to ammonium by oxidizing NADH without producing energy and can be present in both assimilated and dissimilated nitrate reduction metabolic processes (Heo et al., 2020; Pandey et al., 2020; Song et al., 2020). It is not excluded the possibility that the produced ammonia may be not completely or timely binding to glutaminase and leaky due to the direct assimilation of intracellular converted nitrite instead of nitrate. This strain might have a leaky assimilatory pathway for nitrite reduction to benefit its neighbors. Perhaps, in such a harsh environment as Antarctica, the cooperative relationship between microorganisms is more conducive to their survival. The nitrate and nitrite reduction to ammonia associated microorganisms and their ecological roles in the Antarctic are worth further study.

Through genome and transcriptome analysis, the gene that reduces nitrate to nitrite in the cell of strain 24S4–2 pointed to *narG*. The nitrate reductase *NarG* reduces nitrate to nitrite by receiving electrons transferred from the respiratory chain, a dissimilated nitrate reduction metabolic process that carries out nitrate respiration (anaerobically) with energy production (Blasco et al., 1992, 2001). We speculate that strain 24S4–2 reduce the accumulated nitrate to nitrite in the cell through dissimilated nitrate reduction (DNRA) when nitrate is present in the environment. However, strain 24S4–2 is an aerobic bacterium, and its nitrate reduction metabolism is not sensitive to oxygen. Although some aerobic bacteria have also been reported to carry out DNRA action, the mechanism is unclear, and it is thought that they may live anaerobically in the presence of nitrate (Samuelsson, 1985; Huang X. et al., 2021). We speculate that intracellular vesicles of strain 24S4–2 play a critical role. The vesicles may provide an anaerobic environment for the reduction of accumulated nitrate to nitrite and energy production through the respiratory chain on the vesicle membrane. A study proposed a vesicle energy regeneration model, implying that vesicles are not passive nitrate storage compartments that accumulate nitrate at the expense of ATP consumption (Beutler et al., 2012). At the same time, nitrate reduction generates a proton dynamic potential (PMF), which can be converted to ATP and pyrophosphate to conserve energy (Salman et al., 2013; van Teeseling et al., 2013). According to our results, the intracellular accumulation of nitrite by strain 24S4–2 in a medium with 3 mM nitrite was higher than the nitrogen source accumulated in a 30 mM nitrate media. This suggests that strain 24S4–2 can not only use nitrite as an energy source but also detoxify it. The nitrite accumulated or converted may be stored in the vesicles to facilitate rapid reduction to ammonia when needed on the one hand and to block the toxicity to cells on the other. In other words, in the absence of the nitrogen source, vesicles may provide not only a nitrogen source but also an energy source, which may be a strategy to adapt to extremely barren environments such as the Antarctic. However, it does need more evidence to prove the function of vesicles. In the future, we will attempt to isolate and obtain vesicles, further investigate their structural characteristics and detect the substances in vesicles. And the function of vesicles and the mechanism of intracellular accumulation of nitrogen source utilization will be explored in depth by stable isotope methods.

**Figure 8:**
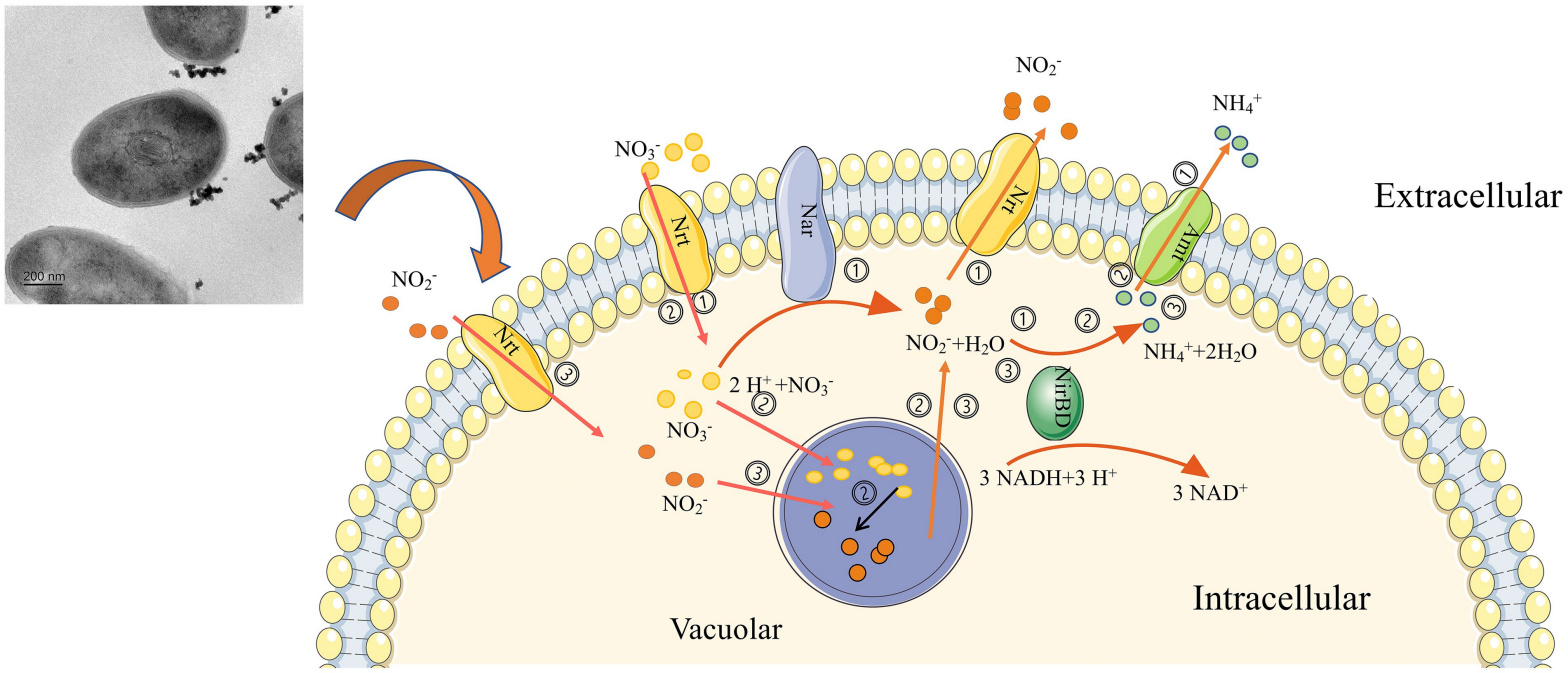
Graphical representation of nitrogen source utilization and accumulation by strain 24S4–2. (i) when nitrate or nitrite is the sole nitrogen source, nitrate is reduced to nitrite and ammonium by strain 24S4–2 and released to the extracellular, (ii) when nitrate is the sole nitrogen source, nitrate is accumulated in the vesicles by 24S4–2, and the accumulated nitrate is also reduced to nitrite, and when the external nitrogen source is absent, the accumulated nitrogen source is reduced to ammonium to maintain its life activities, and (iii) when nitrite is the sole nitrogen source, nitrite is accumulated in the vesicles like nitrate for later utilization.

Based on the findings, we predicted that strain 24S4–2 has various nitrogen metabolic pathways (Figure 8), (i) Strain 24S4–2 might produce ammonia from nitrate or nitrite and release it extracellularly, (ii) When nitrate is the sole nitrogen source, 24S4–2 accumulates nitrate in vesicles made up of folded membranous structures. It reduces the nitrate to nitrite, and when the external nitrogen source is absent, the accumulated nitrogen source is reduced to ammonium to maintain its life activities, and (iii) When nitrite is supplied as the sole nitrogen, nitrite is accumulated in the vesicles like nitrate for later utilization.

However, the current evidence from electron microscopy images and intracellular nitrite/nitrate is insufficient alone, and intracellular nitrite/nitrate concentration measurements cannot fully distinguish the intracellular site of DNRA response. The vesicle and its structure will be further explored by isolation and purification in the future to determine the role of vesicles in the DNRA process, and nitrogen source accumulation, the type, source, and destination of intracellularly accumulated nitrogen sources in strain 24S4–2 will be further investigated using stable isotope labeling of nitrogen sources. In addition, the role of nitrate accumulation and reduction mechanism on the Antarctic bacterial community and ecology will be explored through further studies on the mechanism of nitrate accumulation and reduction. And through identifying key genes for both nitrogen accumulation and ammonia production to provide new indicator genes for investigating nitrogen metabolism in the environment.

## 5. Conclusion

*Arthrobacter* sp. 24S4–2, a potential novel species isolated in Antarctica, can convert nitrate and nitrite to ammonia. Furthermore, it can accumulate nitrate and nitrite intracellularly, convert the accumulated nitrate to nitrite, and use the accumulated nitrogen source to grow and produce ammonia under nitrogen-free conditions. The accumulation and conversion of nitrogen sources are thought to be linked to the intracellular vesicle membrane structure and genes *nirB*, *nirD,* and *nasA*. This space–time conversion process of nitrogen source helps the strain to maintain development in the absence of nitrogen supply or a harsh environment. The process may also play an important ecological role, that other bacteria in the environment would benefit from its extracellular nitrogen source secretion and nitrite consumption characteristics.

## Data availability statement

The original contributions presented in the study are included in the article/Supplementary material, further inquiries can be directed to the corresponding author.

## Author contributions

YL: conceptualization, methodology, software, and data curation. YZ: writing – original draft preparation. XP: visualization and investigation. JN: supervision. YH: software and validation. JH: writing – reviewing and editing. All authors contributed to the article and approved the submitted version.

## Funding

This work was supported by the National Science and Technology Fundamental Resources Investigation Program of China (2021FY100900), the National Key Research and Development Program of China (2022YFC2807501), the R&D Infrastructure and Facility Development Program of the Ministry of Science and Technology of the People’s Republic of China (grant no. NIMR-2020–8), the National Natural Science Foundation of China (grant no. 42076230), and the Chinese Polar Scientific Strategy Research Fund (IC201706).

## Conflict of interest

The authors declare that the research was conducted in the absence of any commercial or financial relationships that could be construed as a potential conflict of interest.

## Publisher’s note

All claims expressed in this article are solely those of the authors and do not necessarily represent those of their affiliated organizations, or those of the publisher, the editors and the reviewers. Any product that may be evaluated in this article, or claim that may be made by its manufacturer, is not guaranteed or endorsed by the publisher.

## Supplementary material

The Supplementary material for this article can be found online at: https://www.frontiersin.org/articles/10.3389/fmicb.2023.1040201/full#supplementary-material
