# Supplementary material for: Spatial and temporal conversion of nitrogen using Arthrobacter sp. 24S4–2, a strain obtained from Antarctica
Source: Front Microbiol. 2023 Feb 15;14:1040201. doi: 10.3389/fmicb.2023.1040201 (PMC9975570; doi:10.3389/fmicb.2023.1040201)
Supplement: Supplementary file 1 [file Data_Sheet_1.docx]

Supplementary Material

**Table S1** RT-qPCR primers

| Gene name | Forward primers | Reverse primers |
| --- | --- | --- |
| *nirB* | CAGTGGCAGAGAAGTACAACC | CCAGCTCCTTCCAGATTTCC |
| *nirD* | TTCCTGCCGTTCCACTTTCC | GTTGAATTTGAACTCGGGCATCC |
| *nasA* | CCGCAATACTTGAACTTGATGAC | CCAGTTCCAGTTCCTCCAC |
| *50S ribosomal L13* | AAGAACGCCAGAACAAGAACGA | CGAGACGACCAAGGACAACG |

**Table S2** O.D.600 of strain 24S4–2 before and after incubation with different oxygen concentrations

| The concentration of O_2_ | 0% | 5% | 20% |
| --- | --- | --- | --- |
| OD_600_(0 h) | 0.111 | 0.114 | 0.112 |
| OD_600_(120 h) | 0.100 | 0.121 | 0.213 |

**Table S3 The growth of strain 24S4-2 at different oxygen concentrations**

| The concentration of O_2_ | 0% | 5% | 20% |
| --- | --- | --- | --- |
| OD_600_(0 h) | 0.111 | 0.114 | 0.112 |
| OD_600_(120 h) | 0.100 | 0.121 | 0.213 |

**Fig. S1** Neighbour-joining phylogenetic tree and the Heatmap of ANI analysis. A, Neighbour-joining phylogenetic tree based on 16S rRNA gene sequences from *Arthrobacter* strain 24S4–2 and related species. The numbers at the branch nodes relate to bootstrap values >50% (1000 replicates). Bar, 0.01 substitutions per site. The outer branches of the evolutionary tree were constructed using the 16S rDNA sequence of *Brevibacterium oceani*. B, Heatmaps show the average nucleotide identity (ANI) value between strain E918T and the reference strain.


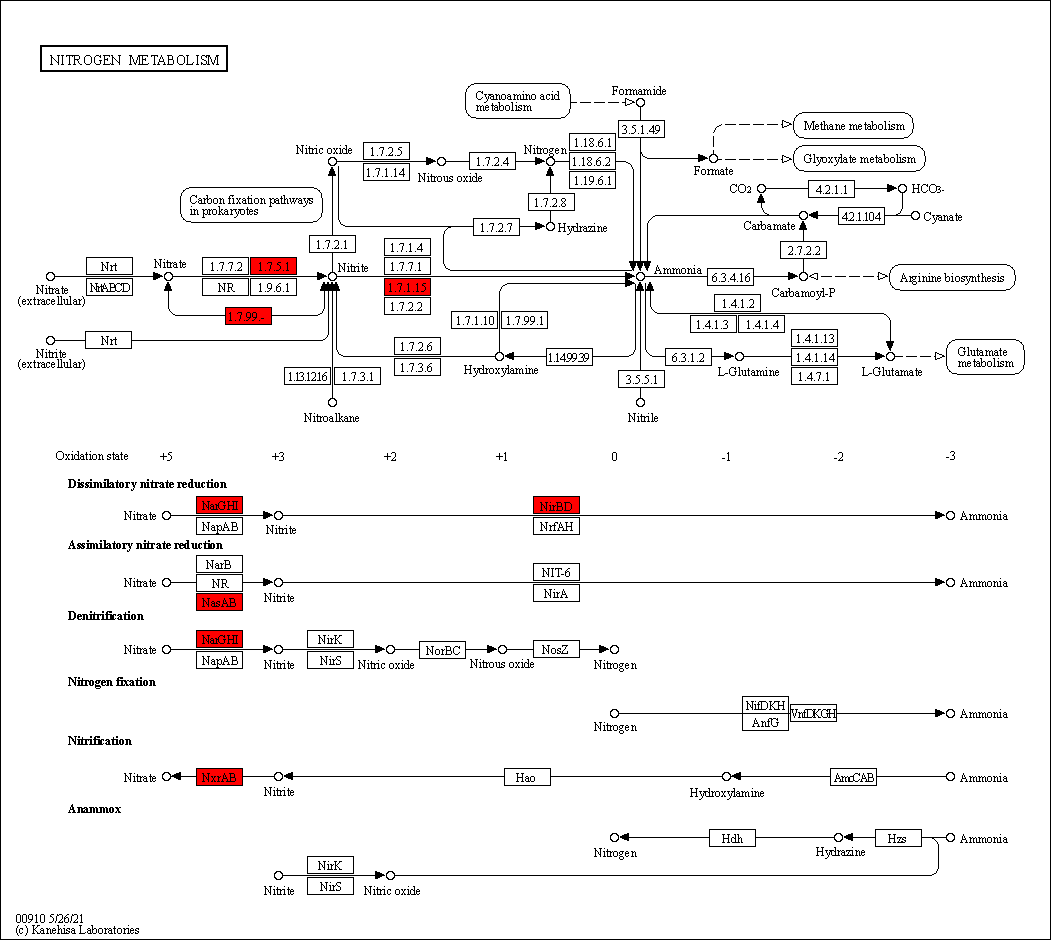


**Fig. S2** Nitrogen metabolism pathway: Nitrogen metabolism-related genes predicted from the genome of strain 24S4–2 are indicated in red boxes.

**Fig. S3** Growth curves of strain 24S4–2 in different nitrogen source media. The black line represents the medium with ammonium as the sole nitrogen source; the gray line represents the medium with nitrate as the sole nitrogen source; the light grey line represents the medium with nitrite as the sole nitrogen source.

**Fig. S4** Growth curves of strain 24S4–2 in different concentrations of nitrite medium.

**Fig. S5** Extracellular nitrite and ammonium concentrations of strain 24S4–2 in different oxygen concentrations (30 mM nitrate medium). There was minimal variation in nitrite production at different oxygen concentrations, whereas 20% oxygen produced more ammonium.

**Fig. S6** The growth curve in nitrogen-free medium, square:indicates prior incubation with nitrate medium for 114h, triangle: indicates strain was incubated with nitrite medium for 114h before the experiment, circle: indicates prior incubation with ammonia salt medium for 114h.

**Fig. S7** Elemental study of ultrathin sections of strain 24S4–2 using transmission electron microscopy. A, elemental analysis of the region of vesicle-like structures in nitrate medium. B, elemental analysis of the non-vesicle region in nitrate medium.
